# Bio-Composite Films Based on Carboxymethyl Chitosan Incorporated with Calcium Oxide: Synthesis and Antimicrobial Activity

**DOI:** 10.3390/polym16172393

**Published:** 2024-08-23

**Authors:** Sarinthip Thanakkasaranee, Pornchai Rachtanapun, Chitsiri Rachtanapun, Thidarat Kanthiya, Gopinath Kasi, Sarana Rose Sommano, Kittisak Jantanasakulwong, Jongchul Seo

**Affiliations:** 1 Division of Packaging Technology, School of Agro-Industry, Faculty of Agro-Industry, Chiang Mai University, Mae-Hea, Mueang, Chiang Mai 50100, Thailand; pornchai.r@cmu.ac.th (P.R.); thidaratkanthiya05@gmail.com (T.K.); gopiscientist@gmail.com (G.K.); kittisak.jan@cmu.ac.th (K.J.); 2 Center of Excellence in Agro Bio-Circular-Green Industry, Faculty of Agro-Industry, Chiang Mai University, Chiang Mai 50100, Thailand; sarana.s@cmu.ac.th (S.R.S.); 3 Department of Food Science and Technology, Faculty of Agro-Industry, Kasetsart University, Bangkok 10900, Thailand; chitsiri.t@ku.th (C.R.); 4 Center for Advanced Studies for Agriculture and Food, Kasetsart University, Bangkok 10900, Thailand; 5 Office of Research Administration, Chiang Mai University, Chiang Mai 50200, Thailand; 6 Department of Plant and Soil Sciences, Faculty of Agriculture, Chiang Mai University, Chiang Mai 50100, Thailand; 7 Department of Packaging, Yonsei University, 1 Yonseidae-gil, Wonju-si 26493, Gangwon-do, Republic of Korea; jcseo@yonsei.ac.kr (J.S.)

**Keywords:** carboxymethyl chitosan (CMCH), calcium oxide (CaO), antimicrobial activity, hydrophilicity, synthesis, carboxymethylation, co-precipitation, polyethylene glycol, active packaging film

## Abstract

The utilization of biopolymers incorporated with antimicrobial agents is extremely interesting in the development of environmentally friendly functional materials for food packaging and other applications. In this study, the effect of calcium oxide (CaO) on the morphological, mechanical, thermal, and hydrophilic properties as well as the antimicrobial activity of carboxymethyl chitosan (CMCH) bio-composite films was investigated. The CMCH was synthesized from shrimp chitosan through carboxymethylation, whereas the CaO was synthesized via a co-precipitation method with polyethylene glycol as a stabilizer. The CMCH-CaO bio-composite films were prepared by the addition of synthesized CaO into the synthesized CMCH using a facile solution casting method. As confirmed by XRD and SEM, the synthesized CaO has a cubic shape, with an average crystalline size of 25.84 nm. The synthesized CaO exhibited excellent antimicrobial activity against *Escherichia coli* (*E. coli*) and *Staphylococcus aureus* (*S. aureus*) (>99.9% R). The addition of CaO into CMCH improved the mechanical and hydrophobic properties of the CMCH-CaO films. However, it resulted in a slight decrease in thermal stability. Notably, the CMCH-CaO10% films exhibited exceptional antimicrobial activity against *E. coli* (98.8% R) and *S. aureus* (91.8% R). As a result, such bio-composite films can be applied as an active packaging material for fruit, vegetable, or meat products.

## 1. Introduction

Generally, food and agriculture products are naturally perishable and need protection from contamination of microorganisms (bacteria, yeast, and fungi) in the air during preparation, storage, and distribution to give them a desired shelf life. These microorganisms can deteriorate the flavor, odor, and texture of foods by undesirable reactions, in which the growth of these microorganisms in such products leads to food-borne illness [1]. In addition, due to the ongoing worldwide pandemic of COVID-19, the food processing industries have faced severe threats to food safety and quality. Therefore, food packaging should provide safe, quality, and microorganism-free food products. These requirements drive the development of active packaging materials such as antimicrobial films for food applications which not only maintain the quality but also extend the shelf life of products [2].

Owing to environmental concerns and the disposal issue of non-degradable food packaging waste, which are highlighted by overflowing landfills and polluted marine waters, the demand for the development of antimicrobial packaging based on biopolymers is increasing [3]. Among biopolymers, carboxymethyl chitosan (CMCH) is a biopolymer that has attracted much attention due to its outstanding properties (e.g., non-toxic, water solubility, absorption–retention, antimicrobial activity, and antioxidant) and which has been applied in various applications (e.g., wound dressing [4], drug delivery [5], and cosmetics [6]). CMCH can be prepared by carboxymethylation, in which the –OH and/or NH_2_ groups of chitosan are substituted by –CH_2_COOH groups. Four different forms of CMCH, namely N-CMCH, N,N-CMCH, O-CMCH, and N,O-CMCH, can be synthesized, in which such structures of the CMCH depend on the conditions (including time and temperature) and reagents (such as NaOH concentration and solvent type) used in their synthesis [7]. Among the various CMCH types, N,O-CMCH is noteworthy because it is an amphoteric chitosan derivative that dissolves easily in water. Because it is a chitosan derivative, the CMCH also retains the characteristic of chitosan viz. good film-forming properties [8]. Thus, CMCH has the potential to be used as a base film in the development of antimicrobial films for food packaging applications. As previously reported, the mechanical strength and flexibility of rice starch film were enhanced after adding CMCH to a rice starch matrix [9]. However, the antimicrobial activity of pristine CMCH may not be enough for some foodstuffs. Thus, the enhancement of the antimicrobial activity of antimicrobial films based on CMCH is required, which can be achieved by incorporating antimicrobial agents. Metal oxides show potential as antimicrobial agents for a wide range of microorganisms. Their positively charged surfaces can interact with negatively charged bacterial surfaces, leading to damage to the bacterial cell membranes, leakage of internal contents, and the death of the bacteria [10,11]. Among metal oxides, CaO is of particular interest because it is regarded as a biocompatible material and non-toxic [12], which has been used in endodontic [13], pharmaceutical [14], and biosensor applications [15]. In addition, the synthesis of CaO has a lower cost compared to the synthesis of silver or gold particles as an active antimicrobial agent. Park et al. (2021) found that CaO obtained from calcined oyster shells presented antimicrobial potency against both *Escherichia coli* (*E. coli*) and *Staphylococcus aureus* (*S. aureus*) [16]. Consequently, CaO could be potentially used as an antimicrobial agent in the development of antimicrobial films based on CMCH and CaO. 

In this study, we aimed to enhance the mechanical, hydrophilic, and antimicrobial properties of CMCH-based active packaging films to broaden their use for perishable foods. This was achieved through the synthesis of CaO and CMCH, and the fabrication of CMCH-CaO bio-composite films using simple and facile methods. The morphological, thermal, mechanical, water contact angle, and antimicrobial activity of the synthesized CaO, CMCH, and CMCH-CaO bio-composite films were investigated. CMCH-CaO bio-composite films with excellent antimicrobial efficiency, and the effect of CaO content on their mechanical, hydrophilic, and antimicrobial properties, have not been reported prior.

## 2. Materials and Methods

### 2.1. Materials

Shrimp chitosan (food grade, SSA190/3K8KF) was purchased from Marine Bio Resources Co., Ltd. (Samut Sakhon, Thailand). Acetic acid glacial anhydrous was purchased from Merck & Co., Inc. (Darmstadt, Germany). Sodium hydroxide (NaOH) was purchased from RCI Labscan Ltd. (Bangkok, Thailand). Ethanol, methanol, and glycerol were purchased from Northern Chemical Co., Ltd. (Chiang Mai, Thailand). Calcium chloride dihydrate and polyethylene glycol (PEG) were purchased from KemAus (Cherrybrook, NSW, Australia). Monochloroacetic acid was purchased from Sigma-Aldrich (Burlington, MA, USA). All of the chemical agents are analytical reagent grade.

### 2.2. Methods 

#### 2.2.1. Synthesis of Carboxymethyl Chitosan

**Figure 1:**
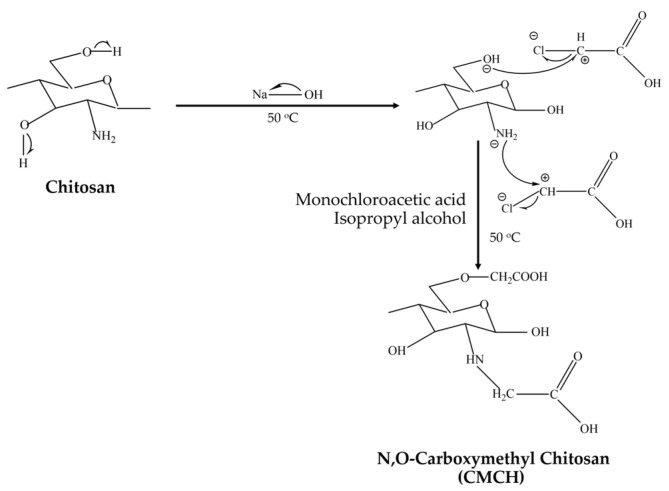
Schematic illustration of N,O-CMCH synthesis.

The N,O-CMCH synthesis is schematically illustrated in Figure 1. Firstly, chitosan was ground and sieved using a Retsch ZM 200 Ultra Centrifugal Mill (En-decotts, London, UK) to achieve particle sizes of 125 µm. Then, 400 mL of 50% (*w*/*v*) sodium hydroxide solution was supplemented with around 25 g of chitosan, followed by 100 mL of isopropanol. This mixture was agitated at 50 °C 1 h, followed by gradual dilution with isopropanol-dissolved monochloroacetic acid. The mixture was placed in an ED 56 drying and heating chamber (Binder Co., Tuttlingen, Germany), heated to 50 °C for 4 h, and filtrated. After filtering, 250 mL of 70% (*v*/*v*) methanol was loaded into the solid and mixed for 10 h. Acetic acid was used to adjust the pH of this combination to 7. This procedure was carried out five times. Subsequently, 250 mL of 95% (*v*/*v*) methanol was added to the solid component, mixed thoroughly for 10 min, and then filtered. The solid was dried at 80 °C for 12 h.

#### 2.2.2. Synthesis of Calcium Oxide

First, 0.5 M of calcium chloride (CaCl_2_) was added to a beaker. Then, 5 wt.% PEG was added and stirred continuously at room temperature. Next, 1 M of NaOH was added dropwise to the mixing solution and stirred at room temperature, followed by stirring at 80 °C for 2 h. Finally, a white precipitate of Ca(OH)_2_ was formed, and the reaction was stopped at pH ≤ 12. The white precipitate was gathered through filtration and washed with distilled water and ethanol before drying at 60 °C. Then, the white precipitate was calcinated at 750 °C for 1 h, and the calcined powders were analyzed and tested.

#### 2.2.3. Preparation of CMCH-CaO Bio-Composite Films

CMCH and CMCH-CaO films were fabricated using a solution casting method. To prepare the CMCH film, 3 g of CMCH was dissolved in 100 mL of distilled water and stirred at 80 °C for 2 h, followed by loading glycerol (as plasticizer). For CMCH-CaO films, CaO was then individually added to the mixture at 1, 5, and 10% of CMCH and stirred for 2 h. Such samples were then degassed, poured onto Petri dishes, and dried at 45 °C for 24 h.

### 2.3. Characterizations

#### 2.3.1. Chemical Structure

A Fourier transform infrared 4700 (FTIR) spectroscopy (Jasco Corp., Tokyo, Japan) was used to analyze the chemical structure of synthesized CMCH powder, synthesized CaO, pristine CMCH film, and CMCH-CaO films.

#### 2.3.2. Morphology and Metal Composition

Morphology was analyzed using a Leica S8 APO Greenough stereo microscope (Leica Microsystems, Wetzlar, Germany) and a JSM IT800LV Scanning Electron Microscope (SEM) (JEOL Ltd., Boston, MA, USA). The element was analyzed using a SEM with energy-dispersive X-ray spectroscopy (EDS). 

#### 2.3.3. Crystal Structure

A Rigaku Miniflex X-ray diffractometer (XRD) (Rigaku mini flex, Tokyo, Japan) was used to analyze the chemical structure of synthesized CaO. A primary beam Cu Kα radiation (λ = 1.541838 Å) was used and the 2θ angle of the diffractometer was gradated from 10 to 90°.

#### 2.3.4. Thermal Stability

Thermal stability was measured using a Mettler–Toledo STARe system TGA/DSC3+ thermogravimetric analyzer (Mettler-Toledo, Greifensee, Switzerland). Approximately 10 mg of the sample was tested under a nitrogen atmosphere (20 mL/min) from 0 to 900 °C at a heating rate of 10 °C/min.

#### 2.3.5. Mechanical Properties 

Tensile strength and elongation at break were measured using a Hounsfield H1KS testing machine (Hounfield Test Equipment, Surrey, UK). The specimen and crosshead speed were prepared and set according to JISK-6251-7. Before measurement, samples were controlled at 25 °C and 50% RH for 48 h. 

#### 2.3.6. Hydrophilic Property

A DSA30B Drop Shape Analyzer (KRÜSS, Hamburg, Germany) was used to measure the water contact angles. Measurements were performed by placing a droplet of water on the samples at 25 °C. The contact angles of the droplet were measured at five distinct locations on each sample, and the results were averaged.

#### 2.3.7. Antimicrobial Activity

The antimicrobial activity of synthesized CaO was evaluated against *Escherichia coli* (*E. coli*) (MTCC 40) and *Staphylococcus aureus* (*S. aureus*) (MTCC 96) using the broth dilution method. *E. coli* and *S. aureus* bacteria were grown on MacConkey and tryptic soy agar plates, respectively, and incubated at 37 °C for 24 h. A single colony was inoculated into 10 mL of nutrient or tryptic soy broth and incubated at 37 °C for 24 h. For the control, 1 mL of bacterial culture was mixed with 1 mL of sterilized DI water in a test tube. In the test samples, 1 mL of bacterial culture was combined with 1 mL of 5 mg CaO in a test tube. The samples were incubated in a humid chamber at 37 ± 1 °C for 24 h. After incubation, the bacterial suspensions were subjected to serial dilution and then loaded on agar plates. These plates were incubated at 37 °C for 48 h and the viable bacterial count was determined. The antimicrobial activity (R) was calculated using the following equation:

$$R\;(\%)=\frac{B-C}{B}\times \;100,$$

where *B* and *C* are the numbers of colony-forming units (cfu) of the viable microbial cells for the control sample (bacterial culture mixed with sterile DI water) and test sample (bacterial culture mixed with CaO) after 24 h, respectively. The tests were performed in triplicate.

The antimicrobial activity of the CMCH and CMCH-CaO films was evaluated against selected microorganisms, like *E. coli* (Gram-negative) and *S. aureus* (Gram-positive), as a model bacterium for perishable food packaging, according to the Japan Industrial Standards (JIS) Z 2801:2000. The *E. coli* and *S. aureus* bacteria were grown on MacConkey and tryptic soy agar plates, respectively, and incubated at 37 °C for 24 h. A single colony was inoculated into 10 mL of nutrient or tryptic soy broth and incubated at 37 °C for 24 h. The surfaces of both control and test samples were sterilized with UV exposure for 15 min. The microbial inoculum (0.4 mL) was loaded onto the test films (50 mm × 50 mm), which were then covered with sterile films (low-density polyethylene, LDPE) (40 mm × 40 mm). The samples were incubated at 37 °C for 24 h. After incubation, each sample was rinsed with 10 mL of phosphate-buffered saline. The bacterial suspensions were subjected to serial dilution and then loaded on agar plates. These plates were incubated at 37 °C for 48 h and the viable bacterial count was determined. The antimicrobial activity (R) was calculated using the following equation:

$$R\;(\%)=\frac{B-C}{B}\times \;100,$$

where *B* and *C* are the numbers of colony-forming units (cfu) of the viable microbial cells for the control sample (LDPE) and test samples (CMCH film, CMCH-CaO1%, CMCH-CaO5%, and CMCH-CaO10% bio-composite films) after 24 h, respectively. The tests were performed in triplicate.

## 3. Results and Discussion

### 3.1. Chemical Structure of Synthesized CMCH and Synthesized CaO

**Figure 2:**
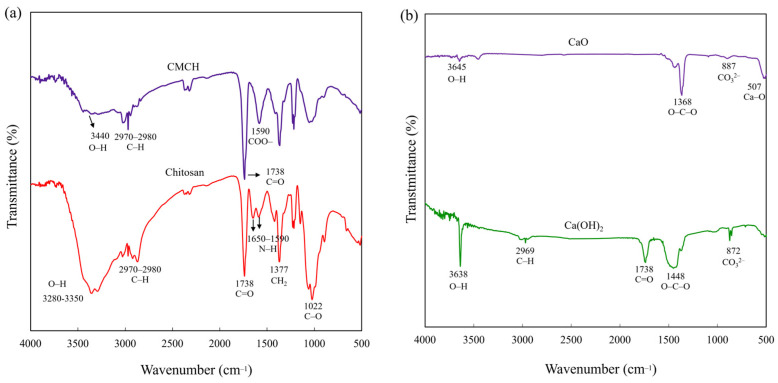
FT-IR spectra: (**a**) Chitosan and synthesized CMCH, and (**b**) Ca(OH)_2_ and synthesized CaO.

The chemical structures of the chitosan, synthesized CMCH, Ca(OH)_2_, and synthesized CaO were characterized using FTIR spectroscopy. Chitosan has characteristic peaks at 3280–3350, 2970–2980, 1738, 1590–1650, 1377, and 1022 cm^−1^, corresponding to O–H, C–H, C=O, N–H, CH_2_, and C–O, respectively [17]. CMCH has characteristic peaks similar to chitosan. Nevertheless, the bimodal peak of N–H (1590–1650 cm^−1^) was merged into the single strong peak of COO^−^ (1590 cm^−1^) because the COO^−^ group overlapped with the original N–H bond of chitosan. This confirmed that the chitosan had successfully converted to CMCH (Figure 2a) [18,19]. Ca(OH)_2_ displayed peaks at 3638 cm^−1^ for bulk OH− groups, 2669 cm^−1^ for C–H of PEG as a surfactant, 1738 cm^−1^ related to C=O from CO_2_ adsorption from the atmosphere, 1448 cm^−1^ for stretching vibration between O–C–O (carbonation of Ca(OH)_2_), 872 cm^−1^ for the carbonate (CO_3_^2−^) group because of fast carbonation of the characteristic CaO surface, and near 500 cm^−1^ corresponding to Ca–O. CaO showed peaks similar to the Ca(OH)_2_. However, the peak intensity of O–H decreased, whereas the peak intensity of Ca–O at 507 cm^−1^ [20] increased, and the C–H of PEG was absent after calcination process (Figure 2b). This implies that the Ca(OH)_2_ precursor was converted to CaO by a calcination process at 750 °C for 1 h. 

### 3.2. Morphology of Synthesized CMCH and Synthesized CaO

**Figure 3:**
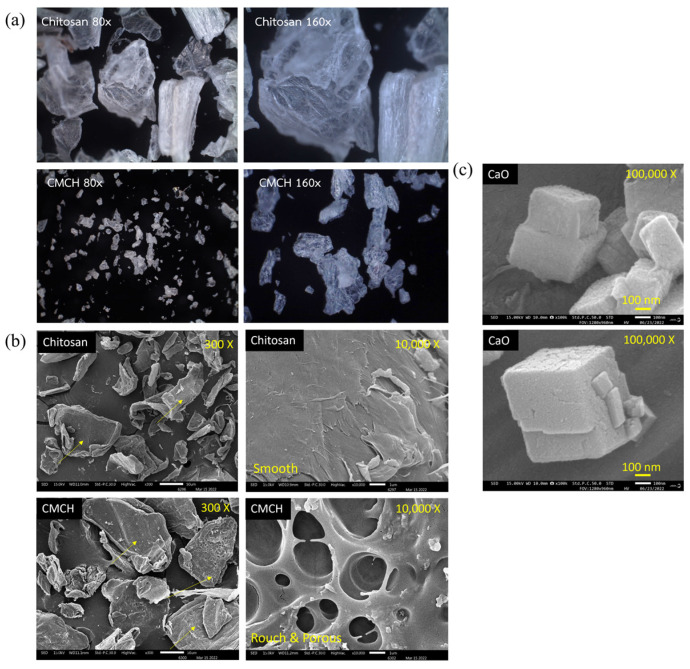
Morphology and physical appearance: (**a**) Stereo microscope images of chitosan and synthesized CMCH, (**b**) SEM images of chitosan and synthesized CMCH, and (**c**) SEM images of synthesized CaO particles.

The morphology of chitosan and synthesized CMCH was analyzed using a stereomicroscope and SEM. The chitosan has an irregular shape, is opaque, and has a relatively smooth surface, while CMCH has a relatively transparent and rougher surface compared to that of chitosan (Figure 3a). Likewise, SEM images showed that the chitosan has a relatively smooth surface, while CMCH has a relatively rough surface and partially porous surface (Figure 3b). This is due to the random damages of the chitosan surface and the weakening of the chitosan structure affected by the strong base (NaOH) used during treatment. Particularly, the partially porous surface appeared in regions where the chitosan surface was most severely damaged, which is the characteristic nature of carbohydrate polymers (chitosan, starch, etc.) after treatment with strong NaOH, leading to a reduction in chitosan crystallinity. This is in agreement with the previous studies [18,21]. Figure 3c shows the morphology of synthesized CaO, which was analyzed using SEM. The synthesized CaO showed a cubic shape, in which the particle size of CaO was approximately 150–400 nm. In general, the particle size and shape of metal oxide are dependent on the synthesis method, type or source of precursor, and synthesis conditions (e.g., stirring time, reaction temperature, calcination temperature/time, etc.). Habte et al. (2020) synthesized CaO nanoparticles from raw eggshells by initially washing, grinding, sieving, and treating them with hydrochloric acid, adding NaOH to the solution to obtain CaCl_2_, and drying them before calcinating at 900 °C for 1 h. They obtained CaO nanoparticles with almost spherical shapes and sizes of 50–198 nm [22]. Likewise, Thakur et al. (2021) synthesized CaO nanoparticles from mollusk shells by initially heating them at 700 °C and crushing them. They then added NaOH to the solution to obtain CaCl_2_, which was filtered and treated with Na_2_CO_3_ to form CaCO_3_. The CaCO_3_ was autoclaved at 200 °C for 2 h, followed by filtration, washing, and final calcination at 900 °C for 2 h. They obtained cubic-shaped CaO nanoparticles with sizes ranging from 1–2 μm [23]. Compared to their synthesis, our approach was facile, using a lower temperature and a process with a shorter time, although this resulted in slightly larger particle sizes (>100 nm). 

### 3.3. XRD Pattern and Elements of Synthesized CaO

**Figure 4:**
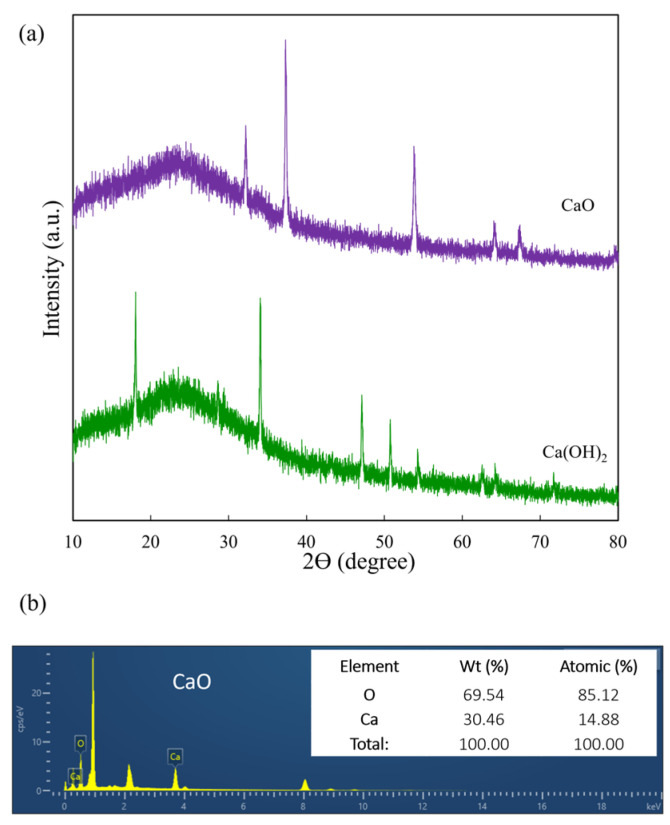
XRD patterns (**a**) and elemental composition (**b**) of synthesized CaO.

XRD patterns of the Ca(OH)_2_ and synthesized CaO are shown in Figure 4a. The Ca(OH)_2_ showed the characteristic peaks at 2θ = 18.1, 28.7, 34.1, 47.1, 50.8, 54.3, 62.8, and 64.2, corresponding to the (001), (100), (101), (102), (110), (111), (201), and (112) planes, respectively. The distinctive peaks were aligned with the standard ICDD database (#00-004-0733). Similar patterns of Ca(OH)_2_ have been reported in multiple literature sources [24,25]. Obviously, after calcination at 750 °C for 1 h. The XRD patterns of CaO showed characteristic peaks at 2θ = 32.2, 37.4, 53.9, 64.3, and 67.5°, matching the crystal planes (111), (200), (220), (311), and (222), respectively. The peaks identified were confirmed using the standard ICDD database (#00-004-0777), demonstrating that no other components were observed and the peaks of Ca(OH)_2_ did not exist, which confirms that the phase of Ca(OH)_2_ had completely changed after the calcination process, which is consistent with the FT-IR result. Similar XRD patterns for CaO have been reported in the literature [26]. Notably, Ca(OH)_2_ has an average crystallite size of 43.38 nm, whereas CaO has an average crystallite size of 25.84 nm. In addition, SEM-EDX was used to analyze the element of synthesized CaO. As shown in Figure 4b, the dominant elements were Ca (14.9%) and O (85.1%). 

### 3.4. Chemical Structures of CMCH-CaO Bio-Composite Films

**Figure 5:**
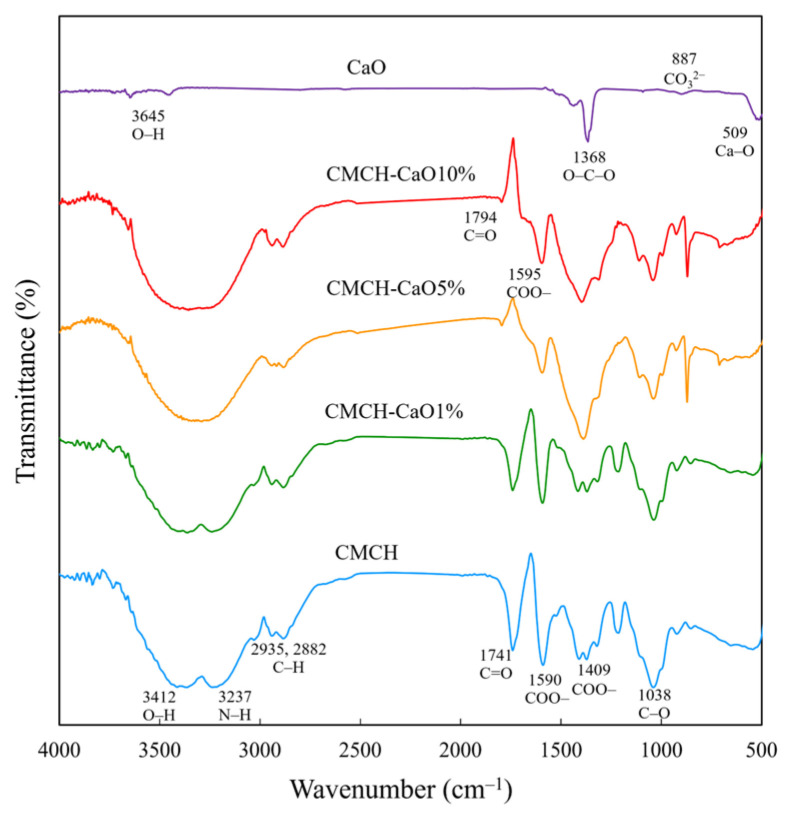
FT-IR spectra of CaO, CMCH, and CMCH-CaO bio-composite films.

The FT-IR spectra of the pristine CMCH and CMCH-CaO films are shown in Figure 5. The pristine CMCH exhibited characteristic peaks at 3412, 3237, 2935–2882, 1740, 1590, 1409, and 1038 cm^−1^, relating to the O–H, N–H, C–H, C=O, COO– group overlapped with the original N–H bond of chitosan, and C–O, respectively. From the overall peak shape, the addition of CaO did not cause major changes to the main structure of CMCH, indicating a physical interaction between CaO and CMCH. The Ca–O peak at 509 cm^−1^ was observed for the CMCH-CaO films with higher CaO contents (5, 10%), indicating that CaO had indeed been added to the CMCH. In addition, the strong peak was observed at 867 cm^−1^, assigned to C–O of CO_3_^2−^ on the surface of CaO particles that dispersed on the film surface. In general, the surface of CaO particles easily interacted with CO_2_ and H_2_O molecules, partially transforming to CaCO_3_ via carbonation and to the Ca(OH)_2_ form via hydration, respectively [27,28]. Additionally, a strong peak was detected at 867 cm^−1^, assigning to C–O of CO_3_^2−^ on the surface of CaO particles that dispersed on the film surface. 

### 3.5. Morphology of CMCH-CaO Bio-Composite Films

**Figure 6:**
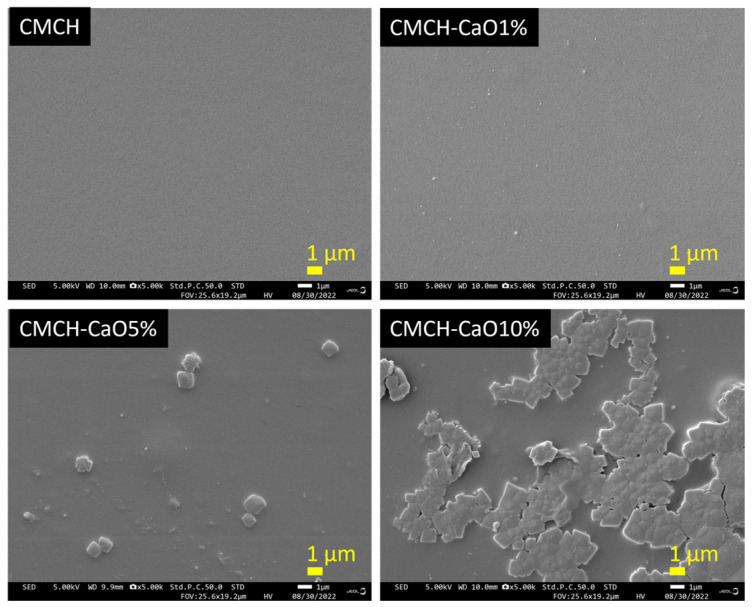
SEM images of CMCH and CMCH-CaO bio-composite films.

The surface morphology of pristine CMCH and CMCH-CaO films are shown in Figure 6. The pristine CMCH film showed a smooth surface, whereas the CMCH-CaO1% film showed a slightly rough surface and good dispersion of small CaO particles in the CMCH matrix. At 5% CaO content, the film had both small and large CaO particles, as well as a rougher surface than the CMCH-CaO1% film. However, the dispersion of CaO particles in the CMCH-CaO 5% film was still good. Thus, the mechanical properties of CMCH films could be improved by adding 1–5% CaO, due to their good dispersion. On the contrary, at 10% CaO content, the film’s surface became rough due to the large agglomeration of CaO, suggesting poor dispersion of CaO in the CMCH matrix. Generally, smaller metal and metal oxide particles have a greater surface area compared to their volume, inducing their tendency to agglomerate. In addition, the degree of agglomeration rises with the addition of more particle content. This aggregation diminishes the effectiveness of particles within the polymer matrix, ultimately leading to inferior properties of the composites [29,30]. As shown in SEM images, the CMCH-CaO 10% bio-composite film exhibited large agglomeration and poor dispersion, which could have resulted in poor mechanical properties.

### 3.6. Thermal Stability of CMCH-CaO Bio-Composite Films

**Figure 7:**
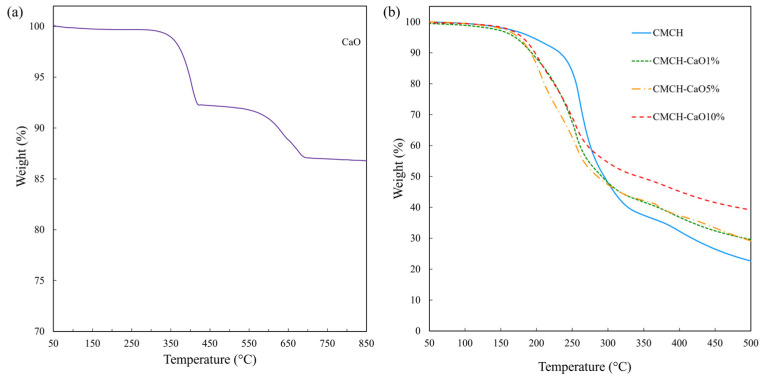
TGA thermograms of (**a**) CaO and (**b**) CMCH-CaO bio-composite films.

Thermal stability refers to a material’s ability to withstand heat without breaking down. The maximum usage temperature is the highest temperature at which a material can be exposed to heat without undergoing degradation [31]. As shown in Figure 7a, the synthesized CaO exhibited two-step decomposition from 300 to 400 °C and from 500 to 700 °C. The initial weight loss was related to the disappearance of water absorbed on the surface of CaO, whereas the second weight loss corresponded to the decomposition of CO_2_ absorbed on the surface of CaO. Remarkably, the thermal stability of CaO was relatively high. This result aligns with previous research [32]. The thermal stability of the CMCH and CMCH-CaO bio-composite films are shown in Figure 7b. The CMCH films exhibited a two-step decomposition process within the temperature ranges of 60–100 °C and 200–400 °C, attributed to the evaporation of residual water as well as the breakdown of hydroxymethyl and amine groups in CMCH [33]. The CMCH-CaO bio-composite films exhibited thermal decomposition behavior similar to that of CMCH film. However, the addition of CaO into CMCH resulted in a slight deterioration in their thermal stability due to low interfacial adhesion between unmodified CaO particles and the CMCH matrix. As a result, lower energy can initially break the polymer chain at the interface during heating, leading to a slight reduction in the thermal stability of the bio-composite films [34,35]. However, most perishable food packaging films, such as wrapped films for meat products, are used at temperatures below 25 °C. Consequently, these bio-composite films can endure such low temperatures, making them suitable for this type of food packaging application.

### 3.7. Mechanical Properties of CMCH-CaO Bio-Composite Films

The effect of CaO content on tensile strength and elongation at the break of the CMCH-CaO bio-composite films is shown in Table 1. The CMCH films showed tensile strength and elongation at break at 5.3 MPa and 76.0%, respectively. The addition of CaO particles into the CMCH resulted in a change in mechanical properties depending on CaO contents. At the low contents of CaO (at 1% and 5% CaO), the tensile strength of films was enhanced by 2–3 times compared to that of the pristine CMCH film. On the contrary, when adding a high CaO content (10% CaO), the tensile strength of the CMCH-CaO bio-composite film decreased. The elongation at break of the CMCH-CaO bio-composite films also depended on the CaO contents. The addition of 5% CaO resulted in a greater improvement of elongation at break compared to the pristine CMCH film. However, loading 1 and 10% CaO content into CMCH decreased the elongation at break. In general, mechanical properties depend on the aggregation or agglomeration and dispersion of particles in the polymer matrix, interfacial interaction between particles and the polymer matrix, compatibility between particles and the polymer matrix, and the reinforcement effect [36,37]. In this study, the films’ mechanical properties were improved by adding 5% CaO. This is related to the reinforcement effect of CaO particles, good dispersion of CaO in the CMCH matrix, and sufficient comparability due to optimal content and their hydrophilic characteristic (hydrophilic particles and hydrophilic polymer). However, at 1% CaO, the content of CaO is relatively low, and when withstanding mechanical force and elongating before breaking, this results in a higher tensile strength but lower elongation at break. However, with 10% CaO, the content is relatively high, leading to agglomeration and poor dispersion of CaO in the CMCH matrix, resulting in a deterioration of the mechanical properties. This mechanical finding aligns with the morphology observations, as demonstrated by the SEM images (Figure 6). However, Sängerlaub et al. (2019) found that the tensile stress remained relatively unchanged, whereas the tensile strain at break decreased with higher CaO concentrations in an LDPE matrix [38]. In addition, Silva et al. (2020) observed that the Young’s Modulus increased, while elongation at break decreased, with the addition of modified CaO nanoparticles to an LDPE matrix [39]. Furthermore, Liu et al. (2019) demonstrated that adding unmodified CaO to polylactic acid (PLA) decreased the tensile strength of the film, whereas the incorporation of modified CaO led to an improvement in tensile strength [40].

### 3.8. Hydrophilicity of CMCH-CaO Bio-Composite Films

**Figure 8:**
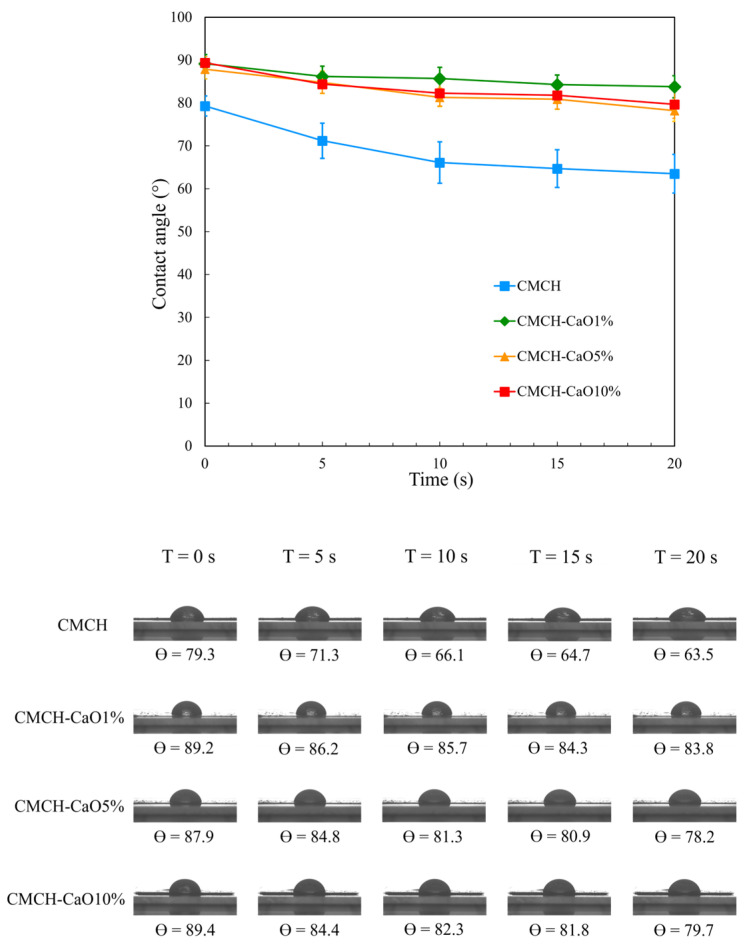
Water contact angles of CMCH and CMCH-CaO bio-composite films.

The hydrophilicity or hydrophobicity of polymer composites is usually investigated using contact angle analysis. The higher water contact angle indicates the greater hydrophobicity in polymer composite materials. As shown in Figure 8, the addition of CaO into CMCH resulted in an increase in the contact angle of films from 79.3° to 89.4° at 0 S. In CMCH-CaO bio-composite films, the water contact angle slightly decreased with rising CaO content. Significantly, CMCH-CaO bio-composite films showed a slight decrease in contact angles over time (0–20 s), whereas the contact angle of the pristine CMCH film decreased rapidly from 0 to 10 s and then declined slightly from 10 to 20 s. This indicates that the hydrophobicity of CMCH-CaO films is improved. The contact angle is mainly influenced by surface morphology and chemical composition [41]. The contact angle of CMCH was lower than that of the CMCH-CaO bio-composite films due to a higher chemical affinity of CMCH to water molecules via a high polar functional group (–COOH, –NH_2,_ and –OH) compared to that of CaO. In addition, the pristine CMCH films have a higher free volume compared to the CMCH-CaO bio-composite films, resulting in a dramatic decrease in contact angle with time. On the contrary, when adding CaO content, CaO particles are hydrophilic materials that tend to increase water absorption and result in a lower water contact angle. However, the dispersion and alignment of CaO within the polymer matrix or film surface can reduce the free volume of the polymer network, which in turn decreases water absorption and leads to a greater contact angle. Consequently, the water contact angle of the CMCH-CaO bio-composite films was higher and decreased at a slower rate over time compared to the pristine films. Further, among CMCH-CaO bio-composite films, the contact angle of films reduced, as CaO content increased. This phenomenon is related to the surface roughness, as the surface of hydrophilic materials becomes rougher, the water contact angle decreases [42]. As shown in the SEM images (Figure 6), the bio-composite films with higher CaO content exhibited a rougher surface. Noticeably, the contact angle result is consistent with morphology findings and also in agreement with earlier studies [43,44].

### 3.9. Antimicrobial Activity of Synthesized CaO and CMCH-CaO Bio-Composite Films

The antimicrobial activity of the synthesized CaO, CMCH, and CMCH-CaO composite films is depicted in Table 2. No detectable colony-forming cells of bacteria at a CaO concentration of only 5 mg/mL for both *E. coli* and *S. aureus* were observed, demonstrating an excellent antimicrobial activity (99.9% R) of the synthesized CaO particles. The antimicrobial activity of CMCH film and bio-composite films was tested compared to LDPE film as a control sample. The number of surviving bacteria for the LDPE film was the highest among all films. The CMCH film showed a lower number of surviving bacteria compared to the LDPE film. The CMCH showed 12.5 and 12.8% R for *E. coli* and *S. aureus*, respectively. As the CaO content rose, the number of surviving bacteria decreased. Notably, the CMCH-CaO bio-composite films demonstrated significantly stronger antimicrobial activity against *E. coli* compared to *S. aureus.* The CMCH-CaO 10% bio-composite film exhibited exceptional antimicrobial activity, effectively inhibiting *E. coli* and *S. aureus* with rates of 98.8 and 91.8% R, respectively. The CMCH-CaO 5% bio-composite film had comparable antimicrobial efficiency against *E. coli* (98.2% R) to the CMCH-CaO 10% film but was less effective against *S. aureus* (88.1% R). This implies that CMCH-CaO bio-composite films were more effective at inhibiting *E. coli* than *S. aureus.* Silva et al. (2020) found that LDPE-CaO films effectively inhibited the growth of *E. coli*. They also observed that the antimicrobial activity of the LDPE-CaO films improved with increasing CaO content. The LDPE-CaO10% showed antimicrobial activity at 30.6 and 81.4% R for smaller CaO nanoparticles (55 nm) and larger CaO nanoparticles (25 nm) [39]. Notably, the CMCH-CaO 5% and CMCH-CaO 10% bio-composite films exhibited greater antimicrobial activity against *E. coli* than the LDPE-CaO 10% film, even though the CaO particles in our synthesized films were larger (150–400 nm).

**Figure 9:**
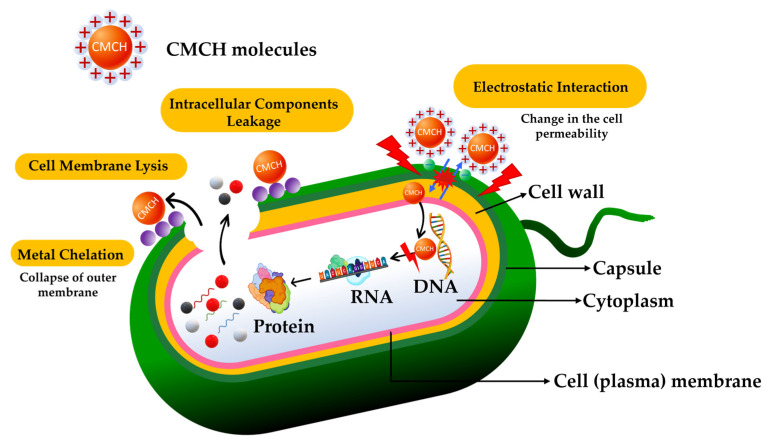
Mechanisms of antimicrobial activity of carboxymethyl chitosan.

The antimicrobial mechanism of CMCH is vitally corresponded to its remained amino group (–NH_2_). The –NH_2_ groups of glucosamine units are protonated to form –NH_3_^+^ when chitosan is dissolved. Their polycationic charge is typically considered the main contributor to its antimicrobial effects, as it causes electrostatic interactions with the microbial cell surfaces [45]. This causes aggregation through charge neutralization and flocculation via a bridging mechanism, ultimately leading to disruption of the cell wall or membrane, leakage of intracellular components, and the inhibition of bacterial growth [46]. In addition, it can create a film on the porins of the cell surface, blocking nutrient exchange and causing microbial cell death. CMCH can also penetrate the cell wall, affecting DNA/RNA and protein synthesis. Moreover, the unprotonated amino groups of CMCH can bind metal ions on the cell surface, disrupting cell walls or membranes, as illustrated in Figure 9. However, the antimicrobial efficiency of the CMCH is dependent on their number of –NH_2_ groups and size. 

**Figure 10:**
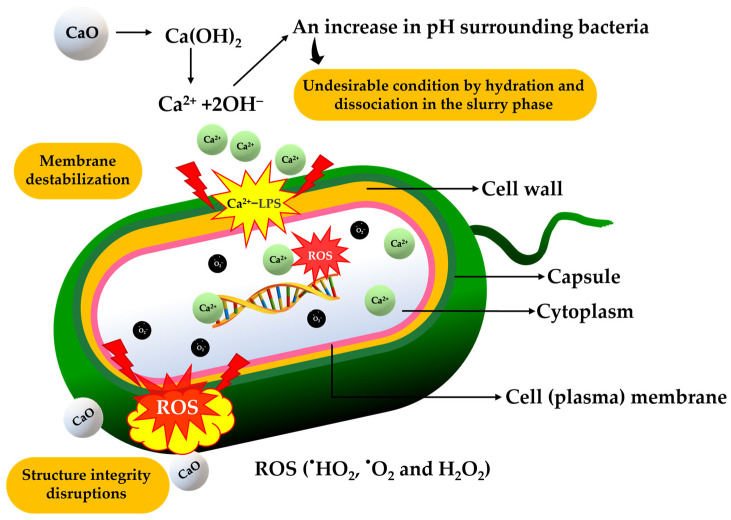
Mechanisms of antimicrobial activity of CaO.

The antimicrobial effectiveness of CaO is influenced by several key factors. CaO raises the pH of the surrounding media, generating a hostile condition for bacteria through its hydration and dissociation. CaO dissociates to release Ca^2+^ ions, interacting with cell surfaces. This interaction can destabilize the membrane and disrupt crucial functions (e.g., membrane transport, energy metabolism, and cell division. Furthermore, CaO produces reactive oxygen species (ROS) like HO^•^, O_2_^•−^, and H_2_O_2_ which further damage bacterial cell membranes. Thus, pH raising, Ca^2+^, and superoxide (O_2_^•−^) ions are factors in their antimicrobial mechanism [47,48], as shown in Figure 10.

Notably, the pristine CMCH films showed low antimicrobial activity against both bacteria. Feng et al. (2021) also indicated a low antimicrobial efficiency in CMCH against *E. coli* and *S. aureus* [49]. Previous research noted that chitosan was typically more effective as an antibacterial agent than CMCH [50]. Significantly, CMCH showed a higher % R against *E. coli* compared to *S. aureus*. This indicates that Gram-negative bacteria are more susceptible to CMCH than Gram-positive bacteria, which could be related to the differences in bacterial cell surface structures. Gram-positive bacteria possess thicker peptidoglycan layers, while Gram-negative bacteria are enriched with lipopolysaccharides (LPSs). LPSs imparts a higher negative charge to Gram-negative bacteria compared to that of Gram-positive bacteria because LPSs are polyanionic molecules that contain abundant negatively charged phosphate groups. The elevated negative charge on Gram-negative bacteria enhances the binding of cationic CMCH (–NH_3_^+^) to their phospholipids, which neutralizes the negative charges from LPSs. This disrupts the outer membrane, facilitating CMCH penetration into the cell membrane, ultimately leading to bacterial cell death [51]. In addition, another antimicrobial mechanism of CMCH (e.g., blocking nutrient exchange, DNA/RNA damages, etc.) may also affect to inhibition of bacteria growth or cell death. 

The incorporation of CaO into the CMCH results in greater antimicrobial action against both bacteria. Remarkedly, the test suggested that Gram-negative bacteria are more susceptible to CaO than Gram-positive bacteria. Alkaline conditions and Ca^2+^ are present when CaO dissociates in a medium (water), generating aggressive conditions for bacteria. Likewise, Ca^2+^ can bind with the abundant negative charges in LPSs of Gram-negative bacteria via electrostatic interaction, resulting in disruption of the outer membrane and leakage of intracellular contents and facilitating Ca^2+^ penetration into the cell membrane. Further, the ROS can also lead to damage to bacterial cell membranes, inhibiting the growth of bacteria, and cell death. Owing to such antimicrobial mechanisms of CMCH and CaO, as well as the difference in the cell wall structure of bacteria, CMCH and CaO more strongly affect *E. coli* than *S. aureus.* Previous studies have also reported that LPS molecules are electrostatically connected via divalent cations (e.g., Mg^2+^ and Ca^2+^), which bind to the anionic phosphate groups in the inner core of the outer membrane for Gram-negative bacteria [52,53]. 

## 4. Conclusions

In this paper, a facile method is used to synthesize CaO and CMCH prior to developing CMCH-CaO bio-composite films. Analyses using a FT-IR, stereoscope, and SEM indicate successful CMCH conversion from chitosan. The XRD and SEM analyses confirm that the synthesized CaO particles are pure, have a cubic shape, and have an average crystallite size of 25.84 nm. Notably, the synthesized CaO exhibited outstanding antimicrobial activity against *E. coli* and *S. aureus*, with >99.9% R. Incorporating CaO into CMCH enhanced the hydrophobicity, tensile strength, elongation at break, and antimicrobial properties of the CMCH-CaO bio-composite films, depending on the CaO content. However, it slightly decreased thermal stability. As the amount of CaO rose, the antimicrobial efficiency of the CMCH-CaO bio-composite films increased. The CMCH-CaO bio-composite films with the highest CaO content presented exceptional antimicrobial efficiency against *E. coli* and *S. aureus*, at 98.8 and 91.8% R, respectively. To achieve over 99% antimicrobial activity, future research needs to focus on modifying the surface of CaO to improve its dispersion, allowing individual CaO particles to effectively interact with bacterial cells. Better dispersion of CaO in the polymer matrix can also enhance the mechanical and thermal stability of the bio-composite films. With such outstanding properties, CMCH-CaO bio-composite films could offer a significant advantage for perishable food applications such as meat, vegetables, or fruit. This finding indicates that CMCH-CaO bio-composite films have potential as an active packaging material (e.g., wrap film, pad, etc.).

## Figures and Tables

**Table 1:** Mechanical properties of CMCH and CMCH-CaO bio-composite films.

Samples	Tensile Strength (MPa)	Elongation at Break (%)
CMCH	5.3 ± 0.4	76.0 ± 3.4
CMCH-CaO1%	15.8 ± 0.8	59.2 ± 1.0
CMCH-CaO5%	13.2 ± 0.8	79.1 ± 2.8
CMCH-CaO10%	4.8 ± 0.2	49.1 ± 1.1

**Table 2:** Antimicrobial activity of synthesized CaO particles and CMCH-CaO bio-composite films.

Samples	*E. coli*		*S. aureus*	
	Viable Cells (CFU/mL)	R (%)	Viable Cells (CFU/mL)	R (%)
CaO	N.D.	≥99.9	N.D.	≥99.9
LDPE	1.06 × 10^9^	-	2.23 × 10^8^	-
CMCH	9.28 × 10^8^	12.5	2.01 × 10^8^	9.82
CMCH-CaO1%	1.87 × 10^8^	82.4	5.56 × 10^7^	75.1
CMCH-CaO5%	1.86 × 10^7^	98.2	2.64 × 10^7^	88.1
CMCH-CaO10%	1.32 × 10^7^	98.8	1.82 × 10^8^	91.8

N.D.: No detectable colony-forming cells of bacteria at a CaO concentration of 5 mg/mL. R: Antimicrobial activity.

## Data Availability

The original contributions presented in the study are included in the article, and further inquiries can be directed to the corresponding authors.
